# High-Resolution Localization Using Distributed MIMO FMCW Radars

**DOI:** 10.3390/s25123579

**Published:** 2025-06-06

**Authors:** Huijea Park, Seungsu Chung, Jaehyun Park, Yang Huang

**Affiliations:** 1 Division of Smart Robot Convergence and Application Engineering, Department of Electronic Engineering, Pukyong National University, Busan 48513, Republic of Korea; gnlwp0620@naver.com (H.P.); 0106325a@naver.com (S.C.); 2 College of Electronic and Information Engineering, Nanjing University of Aeronautics and Astronautics, Nanjing 210016, China; yang.huang.ceie@nuaa.edu.cn

**Keywords:** FMCW MIMO radar, distributed radars, joint range and angle estimation, 2D MUSIC algorithm, coordinate transformation

## Abstract

Due to its fast processing time and robustness against harsh environmental conditions, the frequency modulated continuous waveform (FMCW) multiple-input multiple-output (MIMO) radar is widely used for target localization. For high-accuracy localization, the two-dimensional multiple signal classification (2D MUSIC) algorithm can be applied to signals received by a single FMCW MIMO radar, achieving high-resolution positioning performance. To further enhance estimation accuracy, received signals or MUSIC spectra from multiple FMCW MIMO radars are often collected at a data fusion center and processed coherently. However, this approach increases data communication overhead and implementation complexity. To address these challenges, we propose an efficient high-resolution target localization algorithm. In the proposed method, the target position estimates from multiple FMCW MIMO radars are collected and combined using a weighted averaging approach to determine the target’s position within a unified coordinate system at the data fusion center. We first analyze the achievable resolution in the unified coordinate system, considering the impact of local parameter estimation errors. Based on this analysis, weights are assigned according to the achievable resolution within the unified coordinate framework. Notably, due to the typically limited number of antennas in FMCW MIMO radars, the azimuth angle resolution tends to be relatively lower than the range resolution. As a result, the achievable resolution in the unified coordinate system depends on the placement of each FMCW MIMO radar. The performance of the proposed scheme is validated using both synthetic simulation data and experimentally measured data, demonstrating its effectiveness in real-world scenarios.

## 1. Introduction

Due to its fast processing time and robustness against harsh environmental conditions, the frequency-modulated continuous waveform (FMCW) multiple-input multiple-output (MIMO) radar is widely used for target localization in military and automotive radar sensing and imaging systems [1,2,3,4]. For target localization, the range and cross-range parameters of targets must be estimated. Accordingly, the two-dimensional discrete Fourier transform (2D DFT) algorithm can be applied to the de-ramped MIMO FMCW baseband signal [1,2].

For high-accuracy localization, the 2D multiple signal classification (2D MUSIC) algorithm has been developed. In this method, a covariance matrix of the signal received from a single FMCW MIMO radar is computed, and its subspace decomposition is performed to obtain the 2D MUSIC spectrum [5,6,7,8]. The 2D MUSIC algorithm is widely used for estimating two-dimensional directions of arrival (DOA) [9,10,11,12,13], where elevation and azimuth angles are jointly estimated using a planar array antenna. Because the dimensionality of the 2D MUSIC spectrum is generally high for high-resolution positioning, the computational complexity is significant, particularly in computing the eigenvalue decomposition of the covariance matrix of the received signal [5,6,14]. To mitigate this issue, several approaches have been proposed. In [5], the 2D MUSIC algorithm is combined with an FFT-based parameter estimator to reduce computational complexity. In [14], the Light MUSIC algorithm is introduced to jointly estimate range and angle parameters for automotive radar systems. In [6], a weighted 2D root MUSIC algorithm is developed for joint angle-Doppler estimation. In [7], a reduced-dimension MUSIC algorithm is proposed for near-field source localization, and in [8], a cascade angle estimation algorithm exploiting CAPON and Beam-Space MUSIC is developed.

To further enhance estimation accuracy, multiple distributed FMCW MIMO radars can be utilized [15,16,17]. In [15], two FMCW MIMO radars are spatially distributed to detect a small drone, with the radars connected via fiber-optic links that exhibit low distortion characteristics and minimal propagation loss. In [16,17], signal-coherent fusion algorithms for multiple distributed radars are proposed, where coherent parameters are efficiently estimated to enable the combination of non-coherently de-ramped signals from multiple radar receivers. In [18], a distributed MIMO radar system is developed to mitigate on-board interference and angular ambiguity, employing a compressive sensing method to estimate the DOA of multiple targets. Similarly, in [19], the problem of multiple target localization in a distributed MIMO radar system is formulated and solved using a compressive sensing approach. However, these approaches require excessive communication resources to share and collect the signals measured from multiple radar receivers. To address this limitation, in [20], MUSIC spectra from multiple FMCW MIMO radars are collected at a data fusion center to reduce data communication overhead. In this approach, distributed radars are positioned along a straight line, and the aggregated MUSIC spectrum at the data fusion center is used to estimate the target’s angle and range within a unified coordinate system. Nevertheless, the transmission of MUSIC spectra still demands substantial communication resources. Moreover, the impact of distributed radar placement on localization performance has not been considered.

To address these issues, we propose an efficient high-resolution target localization algorithm using distributed MIMO FMCW radars. Instead of transmitting the raw received signal data or MUSIC spectra to the data fusion center [15,16,17,20], the distributed radars forward target position estimates relative to their own positions and the collected estimates are combined using a weighted averaging approach at the data fusion center. This approach significantly reduces data communication overhead. To effectively combine the local estimates, we first analyze how errors in local parameter estimation (i.e., range and angle) affect target localization errors within a unified coordinate system. Based on this analysis, the local estimates are weighted and averaged to determine the target position at the data fusion center. The weights are assigned according to local parameter estimation errors, which are determined by the achievable parameter resolution within the unified coordinate framework. Notably, localization errors depend not only on errors in range and angle estimation at each radar but also on the estimates themselves. Due to the typically limited number of antennas in FMCW MIMO radars, the azimuth angle resolution tends to be relatively lower than the range resolution. As a result, localization errors in the unified coordinate system are influenced by the placement of each FMCW MIMO radar. By evaluating root-mean-squared-error (RMSE) performance through computer simulations, we analyze the impact of distributed FMCW MIMO radar placement on overall radar performance. Furthermore, by applying the proposed scheme to experimentally measured data, we demonstrate its effectiveness in real-world scenarios. Recent developments in novel antenna array technologies, including reconfigurable intelligent surfaces (RISs) [21] and time-modulated arrays (TMAs) [22], offer promising directions for enhancing distributed radar systems. These technologies could potentially improve signal quality, mitigate multipath effects, and provide adaptive beamforming capabilities in future distributed MIMO radar implementations.

The rest of this paper is organized as follows. In Section 2, we introduce the system model for distributed FMCW MIMO radars and present the reformulation of the received signal to facilitate joint range and angle estimation at each FMCW MIMO radar. In Section 3, we briefly introduce two target localization algorithms: the 2D FFT algorithm and the 2D MUSIC algorithm, where the range and azimuth angle of a target are jointly estimated. Additionally, we discuss how the resolution of range and angle estimation contributes to target localization errors. In Section 4, we propose an efficient high-resolution target localization algorithm. Here, we explain how to determine the target position in a unified coordinate system at the data fusion center using target position estimates obtained from multiple FMCW MIMO radars. In Section 5, we present various simulation results, and in Section 6, we experimentally validate the proposed scheme using real data measured with two W-band FMCW radars. Finally, in Section 7, we provide our conclusions.

## 2. System Model for a Distributed FMCW MIMO Radar System

**Figure 1:**
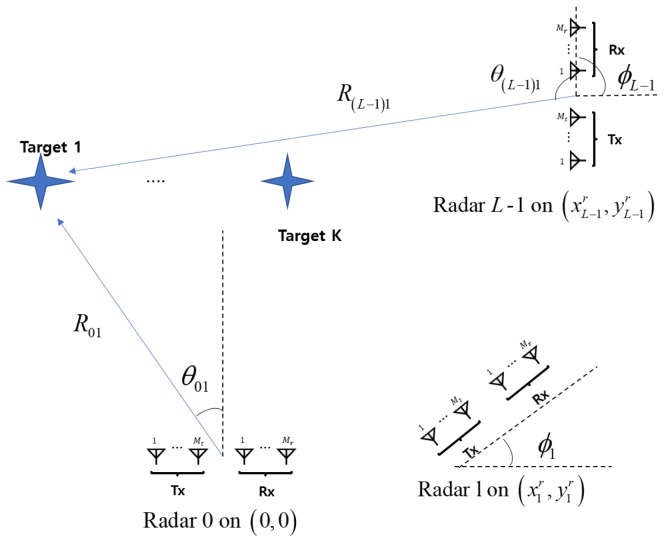
Distributed FMCW MIMO radar system.

In this paper, as shown in Figure 1, we consider the distributed FMCW MIMO radar system, where each FMCW MIMO radar consists of $M_{t}$ number of transmit (Tx) antennas and $M_{r}$ number of receive (Rx) antennas. Here, it is assumed that *L* FMCW MIMO radars are distributed in a given area and the *l*th radar is located at $\left(x_{l}^{r},y_{l}^{r}\right)$, l=0,…,L−1. Without loss of generality, the 0-th radar is set as the reference radar and its location is set as $\left(x_{0}^{r},y_{0}^{r}\right)=\left(0,\;0\right)$. If *K* targets are randomly located at $\left(x_{k}^{t},y_{k}^{t}\right)$, the *k*th target has the angle of $\theta_{lk}$ and the range of $R_{lk}$, with respect to the *l*th FMCW MIMO radar, given as

(1) $$\theta_{lk}=\tan^{-1}\left(\frac{x_{k}^{t}-x_{l}^{r}}{y_{k}^{t}-y_{l}^{r}}\right)+\phi_{l},$$

(2) $$R_{lk}=\sqrt{{\left(x_{k}^{t}-x_{l}^{r}\right)}^{2}+{\left(y_{k}^{t}-y_{l}^{r}\right)}^{2}},$$

where $\phi_{l}$ is the angle by which the *l*th radar is rotated counterclockwise around the *x* axis.

### 2.1. Transmitted/Received Signal Model for a Distributed FMCW MIMO Radar

If the $m_{t}$th Tx antenna element of the the *l*th radar transmits a FMCW signal within a pulse duration $T_{PR}$, the associated FMCW signal can be expressed as

(3) $$s_{l}^{m_{t}}\left(t\right)=\exp\left\{j\left(2\pi \left(f_{c}+\Delta f_{c}\left(m_{t}-1\right)+\Delta f_{d}\left(l-1\right)\right)t+\pi \alpha t^{2}\right)\right\},$$

for $0\;\leq \;t\;\leq \;T_{PR}$, where $f_{c}$ is the carrier frequency and α is the chirp rate. In (3), frequency offset $\Delta f_{c}$ (respectively, $\Delta f_{d}$) is introduced to avoid the intra-radar interference caused by the Tx antennas within each FMCW MIMO radar (respectively, the inter-radar interference caused by the Tx antennas from different FMCW MIMO radars).

When the transmit signal $s_{l}^{m_{t}}\left(t\right)$ is reflected from the *k*th target, the reflected signal is received at each the $m_{r}$th Rx antenna of the *l*th radar, given as

(4) $$r_{l}^{m_{r}}\left(t\right)=\sum_{k=1}^{K}\sum_{m_{t}=1}^{M_{t}}\gamma_{lk}s_{l}^{m_{t}}\left(t-\tau_{lk}^{m_{r}m_{t}}\right)+n_{l}^{m_{r}}\left(t\right),$$

where $n_{l}^{m_{r}}\left(t\right)$ denotes the additive white Gaussian noise. Note that $\tau_{lk}^{m_{r}m_{t}}$ is the propagation time delay and $\gamma_{lk}$ implies the aggregated target reflection coefficient. That is, the antenna gain and the path-loss are aggregated in $\gamma_{lk}$. Then, $\gamma_{lk}$ and $\tau_{lk}^{m_{r}m_{t}}$ can be given as

(5) $$\gamma_{lk}=\frac{G}{{\left(R_{lk}^{m_{t}}\right)}^{2}{\left(R_{lk}^{m_{r}}\right)}^{2}}\approx \frac{G}{{\left(R_{lk}\right)}^{4}}\gamma_{k},$$

and

(6) $$\begin{array}{ccc} \tau_{lk}^{m_{r}m_{t}} & = & \frac{2}{c}\left(\frac{R_{lk}^{m_{t}}+R_{lk}^{m_{r}}}{2}\right)\\ & \approx & \frac{2}{c}\left(R_{lk}+d\left(m-1\right)\sin\theta_{lk}\right), \end{array}$$

where *G* and $\gamma_{k}$ is the antenna gain and the reflection gain of the *k*th target. If it is assumed that there is a far-field distance between the *k*th target and the *i*th radar, ${\left(R_{lk}^{m_{t}}\right)}^{2}{\left(R_{lk}^{m_{r}}\right)}^{2}$ can be approximated as ${\left(R_{lk}\right)}^{4}$. Without loss of generality, $\gamma_{k}$ is modeled as a complex Gaussian random variable (i.e., $\gamma_{k}∼\mathrm{CN}\left(0,\;1\right)$). In (5) and (6), $R_{lk}^{m_{t}}$ (resp., $R_{lk}^{m_{r}}$) is the distance between the $m_{t}$th Tx (resp., the $m_{r}$th Rx) antenna at the *l*th radar and the *k*th target. Here, *d* is the inter-antenna spacing when the virtual antenna array is formed as a linear array by properly locating Tx/Rx antennas [23]. Throughout the paper, it is assumed that d=λ/2, where λ is the wavelength of the FMCW radar waveform. In the last approximation in (6), $R_{lk}$ is the distance between the position of the reference element (i.e., the first element in the virtual array) in the *l*th radar and the *k*th target. Here, we use the virtual element index $m=1,\ldots ,M_{r}M_{t}$ instead of Tx/Rx indices. Then, (6) can be rewritten as

(7) $$\tau_{lk}^{m}=\tau_{lk}+\frac{2}{c}\left(d\left(m-1\right)\sin\theta_{lk}\right),$$

for $m=1,\ldots ,M_{t}M_{r}$ with $\tau_{lk}=2R_{lk}/c$. The received baseband signal at each *l*th radar is then given as

(8) $$\begin{array}{ccc} x_{l}^{m}\left(t\right)\; & \;=\; & \;LP\{r_{l}^{m_{r}*}\left(t\right)s_{l}^{m_{t}}\left(t\right)\}\\ \; & \;=\; & \;\sum_{k=1}^{K}\;\sum_{m_{t}=1}^{M_{t}}\gamma_{lk}\exp\{j(2\pi (f_{c}+\Delta f_{c}\left(m_{t}-1\right)\\ & & +\Delta f_{d}\left(l-1\right))\tau_{lk}^{m}+2\pi \alpha \tau_{lk}^{m}t-\pi \alpha {\left(\tau_{lk}^{m}\right)}^{2})\}+n_{lm}\left(t\right)\\ & \approx & \sum_{k=1}^{K}\gamma_{lk}exp\left\{j\left(2\pi f_{c}\tau_{lk}^{m}+2\pi \alpha \tau_{lk}^{m}t-\pi \alpha {\left(\tau_{lk}^{m}\right)}^{2}\right)\right\}\\ & & +n_{lm}\left(t\right), \end{array}$$

where $n_{lm}\left(t\right)∼\mathrm{CN}\left(0,\sigma_{n}^{2}\right)$ and LP{·} is the low-pass filter output, which passes the beat frequency component of the received signal. Here, the inner product of the Tx waveform and the Rx signal is the de-ramping process. In addition, the last approximation in (8) comes from that $f_{c}\;\gg \;\Delta f_{c}\left(resp,\Delta f_{d}\right)$.

Throughout the paper, we consider the line-of-sight (LoS) components, while neglecting non-LoS (NLoS) components as in [24,25,26]. It is well-justified for practical radar applications because NLoS components are typically 10–20 dB weaker than LoS signals due to path loss and reflection losses.

### 2.2. Transforming Received Signals into a Discrete-Time Signal Matrix

To convert (8) into a discrete time domain signal, $x_{m}^{\left(i\right)}\left(t\right)$ is sampled with the sampling frequency, $f_{s}=1/T_{s}$, given as

(9) $$\begin{array}{ccc} x_{l}^{m}\left[n\right] & \;=\; & x_{l}^{m}\left(nT_{s}\right)\\ & \;\approx \; & \sum_{k=1}^{K}\gamma_{lk}exp\{j(2\pi f_{c}\left(\tau_{lk}+\frac{2}{c}\left(d\left(m-1\right)\sin\theta_{lk}\right)\right)\\ & \;\; & +j2\pi \alpha \left(\tau_{lk}+\frac{2}{c}\left(d\left(m-1\right)\sin\theta_{lk}\right)\right)nT_{s}\}+n_{lm}\left[n\right], \end{array}$$

where $n_{lm}\left[n\right]≜n_{lm}\left(nT_{s}\right)$. Here, $\tau_{lk}^{m}$ in (7) is substituted into (8) and the second order term $\pi \alpha {\left(\tau_{lk}^{m}\right)}^{2}$ in (8) is ignored. Furthermore, for far-field target range, $\tau_{lk}\gg \frac{2}{c}\left(d\left(m-1\right)\sin\theta_{lk}\right)$ and therefore $x_{l}^{m}\left[n\right]$ in (9) can be approximated as

(10) $$\;x_{l}^{m}\left[n\right]\;\approx \;\;\sum_{k=1}^{K}{\bar{\gamma }}_{lk}exp\left\{j2\pi f_{c}\left(\frac{2}{c}\left(d\left(m-1\right)\sin\theta_{lk}\right)\right)+j2\pi \alpha \tau_{lk}nT_{s}\right\},$$

where ${\bar{\gamma }}_{lk}=\gamma_{lk}exp\left\{j2\pi f_{c}\tau_{lk}\right\}$. In this paper, the total *S* FMCW pulses are collected at the receiver and by introducing the pulse index *s*, the discrete signal $x_{l}^{m}\left[n,s\right]$, the *n*th sample of the *s*th FMCW pulses, can then be represented as

(11) $$\begin{array}{ccc} x_{l}^{m}\left[n,s\right] & \approx & x_{l}^{m}\left(nT_{s}\right){|}_{s_{th}pulse}\\ & \approx & \sum_{k=1}^{K}{\bar{\gamma }}_{lk}exp\{j2\pi f_{c}\left(\frac{2}{c}\left(d\left(m-1\right)\sin\theta_{lk}\right)\right)\\ & & +j2\pi \alpha \tau_{lk}\left(sT_{PR}+nT_{s}\right)\}\\ & = & \sum_{k=1}^{K}{\bar{\gamma }}_{lk}exp\{j2\pi f_{c}\left(\frac{2}{c}\left(d\left(m-1\right)\sin\theta_{lk}\right)\right)\\ & & +j2\pi \alpha \tau_{lk}T_{s}n+j2\pi \alpha \tau_{lk}T_{PR}s\} \end{array}$$

Then, from $exp\{j2\pi f_{c}(2/c\left(d\left(m-1\right)\sin\theta_{lk}\right\}$ in (11), by introducing the array response vector $a(\theta_{lk})$, $x_{l}^{m}\left[n,s\right]$ can be rewritten as a vector form. Specifically, for a linear array antenna, the array steering vector is given as

(12) $$a\left(\theta_{lk}\right)=\left[\begin{array}{c} 1\\ exp\{2\pi \frac{2d}{\lambda }\sin\theta_{lk}\}\\ \vdots \\ exp\{2\pi \frac{2d(M-1)}{\lambda }\sin\theta_{lk}\} \end{array}\right]$$

and $x_{l}^{m}\left[n,s\right]$ can then be vectorized as

(13) $$\begin{array}{ccc} x_{l}\left[n,s\right]\; & \;=\; & \;\left[\begin{array}{c} x_{l}^{1}\left[n,s\right]\\ \vdots \\ x_{l}^{M}\left[n,s\right] \end{array}\right]\\ \; & \;=\; & \;\sum_{k=1}^{K}{\bar{\gamma }}_{lk}a\left(\theta_{lk}\right)exp\left\{j2\pi \alpha \tau_{lk}T_{s}n+j2\pi \alpha \tau_{lk}T_{PR}s\right\}. \end{array}$$

## 3. Target Localization Algorithm with a Single FMCW MIMO Radar

For the target localization with a single FMCW MIMO radar, we first consider the 2D-FFT based parameter estimation algorithm and investigate how the estimation errors affect the localization error in a unified coordinate. Then, we also present the subspace-based 2D-MUSIC algorithm for the high-resolution parameter estimation.

### 3.1. 2D DFT Based Target Localization and the Resolution Analysis in a Unified Coordinate

Without loss of generality, we consider a single target with $\left(\theta_{l1},R_{l1}\right)$, and then, by stacking $x_{l}^{T}\left[n,s\right]$ for n=1,…,N, we can have

$$\begin{array}{ccc} {\tilde{X}}_{l}\left[s\right] & = & {\bar{\gamma }}_{l1}b\left(\tau_{l1}\right)a^{T}\left(\theta_{l1}\right)exp\left\{j2\pi \alpha \tau_{l1}T_{PR}s\right\}, \end{array}$$

where $b(\tau_{l1})$ is given as

(14) $$\begin{array}{ccc} b(\tau_{l1}) & = & C[1\cdots \exp{\left\{j2\pi \left(\alpha \tau_{l1}\right)\left(N-1\right)T_{s}\right]}^{T}, \end{array}$$

where $C≜\exp\{j2\pi \left(\alpha \tau_{l1}\right)T_{s}\}$. We note that $b(\tau_{l1})$ is the fast-time array response vector associated with $\tau_{l1}$, while $a(\theta_{l1})$ is a spatial response vector associated with $\theta_{l1}$. By applying the 2D DFT to ${\tilde{X}}_{l}\left[s\right]$, we have the following DFT spectrum,

(15) $$\begin{array}{ccc} S(i,j) & = & \sum_{n=1}^{N}\sum_{m=1}^{M}{\left({\tilde{X}}_{l}\left[s\right]\right)}_{n,m}e^{-j2\pi \frac{i}{N}n}e^{-j2\pi \frac{j}{M}m}. \end{array}$$

Then, the range and the angle estimates are given as

(16) $$\begin{array}{c} {\hat{R}}_{l1}=\frac{c}{2k}\left(\frac{\hat{i}}{NT_{s}}\right),\;{\hat{\theta }}_{l1}=\sin^{-1}\left(\frac{\lambda }{d}\left(\frac{\hat{j}}{M}-0.5\right)\right), \end{array}$$

where

(17) $$\begin{array}{ccc} \left(\hat{i},\hat{j}\right) & = & \arg\max|S(i,j)|. \end{array}$$

Then, the target location estimated from the *l*th radar can be given as

(18) $$\begin{array}{ccc} \left({\hat{x}}_{l1}^{t},{\hat{y}}_{l1}^{t}\right) & = & ({\hat{R}}_{l1}\sin{\hat{\theta }}_{l1}+x_{l}^{r},{\hat{R}}_{l1}\cos{\hat{\theta }}_{l1}+y_{l}^{r}). \end{array}$$

The following lemma is useful to understand how the resolution of range and angle estimation contributes on the target localization errors.

**Lemma** **1.** *When ${\hat{R}}_{l1}=R_{l1}+\Delta R_{l}$ and ${\hat{\theta }}_{l1}=\theta_{l1}+\Delta \theta_{l}$, the target location estimated from the lth radar can be given as*

(19) $$\begin{array}{ccc} \left({\hat{x}}_{l1}^{t},{\hat{y}}_{l1}^{t}\right) & = & (x_{l1}^{t}+\Delta x_{l}^{t},y_{l1}^{t}+\Delta y_{l}^{t}), \end{array}$$

*where*

(20) $$\begin{array}{c} \left[\begin{array}{c} \Delta x_{l}^{t}\\ \Delta y_{l}^{t} \end{array}\right]=\left[\begin{array}{cc} \sin\theta_{t} & \cos\theta_{t}\\ \cos\theta_{t} & -\sin\theta_{t} \end{array}\right]\left[\begin{array}{c} \Delta R_{l}\\ R_{tl}\Delta \theta_{l} \end{array}\right]. \end{array}$$

**Proof.**  From (18),

(21) $$\begin{array}{ccc} \;{\hat{x}}_{l1}^{t}-x_{l}^{r} & = & \left(R_{l1}+\Delta R_{l}\right)\sin\left(\theta_{l1}+\Delta \theta_{l}\right)\\ & = & \left(R_{l1}+\Delta R_{l}\right)\left(\sin\left(\theta_{l1}\right)\cos\left(\Delta \theta_{l}\right)+\cos\left(\theta_{l1}\right)\sin\left(\Delta \theta_{l}\right)\right)\\ & \approx & \left(R_{l1}+\Delta R_{l}\right)\left(\sin\left(\theta_{l1}\right)+\Delta \theta_{l}\cos\left(\theta_{l1}\right)\right)\\ \;\; & \;\;\approx \;\; & \;\;R_{l1}\sin\left(\theta_{l1}\right)+\Delta R_{l}\sin\left(\theta_{l1}\right)+R_{l1}\Delta \theta_{l}\cos\left(\theta_{l1}\right). \end{array}$$

Therefore,

(22) $$\begin{array}{c} {\hat{x}}_{l1}^{t}=x_{l1}^{t}+\Delta R_{l}\sin\left(\theta_{l1}\right)+R_{l1}\Delta \theta_{l}\cos\left(\theta_{l1}\right). \end{array}$$

By taking a similar approach to ${\hat{y}}_{l1}^{t}$, we have

(23) $$\begin{array}{c} {\hat{y}}_{l1}^{t}=y_{l1}^{t}+\Delta R_{l}\cos\left(\theta_{l1}\right)-R_{l1}\Delta \theta_{l}\sin\left(\theta_{l1}\right). \end{array}$$

□

We note that the localization errors depend on both the errors in the range/angle estimation at each radar and their estimates themselves. In addition, the errors in the range and the angle estimation at each radar depend on the resolution of range and angle estimation, given as [27]

(24) $$\begin{array}{c} {\mathrm{Res}}_{R}=\frac{c}{2BW},\;{\mathrm{Res}}_{\theta }=\frac{2}{M}. \end{array}$$

That is, $|\Delta R_{l}|\;\leq \;\frac{{\mathrm{Res}}_{R}}{2}=\frac{c}{4BW}$ and $|\Delta \theta_{l}|\;\leq \;\frac{{\mathrm{Res}}_{\theta }}{2}=\frac{1}{M}$.

### 3.2. Subspace-Based 2D MUSIC Algorithm with a Single FMCW MIMO Radar

In the FMCW MIMO radar system, to obtain high-resolution estimates of the ranges and azimuth angles of multiple targets, the subspace-based 2D MUSIC algorithm can be utilized. Specifically, by stacking $x_{l}\left[n,s\right]$ for n=1,…,N in (13), we can have

(25) $$\begin{array}{ccc} {\bar{x}}_{l}\left[s\right] & = & \left[\begin{array}{c} x_{l}\left[1,s\right]\\ \vdots \\ x_{l}\left[N,s\right] \end{array}\right]\\ & = & \sum_{k=1}^{K}{\bar{\gamma }}_{lk}b\left(\tau_{lk}\right)⊗a\left(\theta_{lk}\right)exp\left\{j2\pi \alpha \tau_{lk}T_{PR}s\right\}, \end{array}$$

where the notation ⊗ is a Kronecker product operator. By concatenating ${\bar{x}}_{l}\left[s\right]$ for s=1,…,S, we have discrete time de-ramped signals as

(26) $$X_{l}=\left[{\bar{x}}_{l}\left[1\right],\cdots ,{\bar{x}}_{l}\left[S\right]\right]$$

The sample covariance matrix of $X_{l}$ is then computed from (26) and its eigenvalue decomposition (EVD) is then given as

(27) $$\begin{array}{cc} {\bar{R}}_{l}=\frac{1}{S}X_{l}X_{l}^{H}=\left[\begin{array}{cc} E_{ls} & E_{ln} \end{array}\right] & \left[\begin{array}{cc} \Lambda_{ls} & 0\\ 0 & \Lambda_{ln} \end{array}\right]{\left[\begin{array}{cc} E_{ls} & E_{ns} \end{array}\right]}^{H}, \end{array}$$

where $E_{ls}\in C^{MN\times K}$ is the matrix whose columns consist of the eigenvectors that span the signal subspace of ${\bar{R}}_{l}$. In addition, the columns $E_{ns}\in C^{MN\times (MN-K)}$ are the eigenvectors that span the noise subspace of ${\bar{R}}_{l}$. Here, $\Lambda_{ls}\in C^{K\times K}$ and $\Lambda_{ns}\in C^{\left(MN-K)\times (MN-K\right)}$ are the diagonal matrices whose diagonal elements consist of the (K,MN−K) eigenvalues of ${\bar{R}}_{l}$. Note that the columns of $E_{ns}$ are orthogonal to those of $E_{ls}$ (equivalently, the signal subspace spanned by the columns of $X_{l}$ in (26)). Accordingly, from (14) and (25), we define f(τ,θ) as

(28) f(τ,θ)≜b(τ)⊗a(θ).

Then, by letting the cost function $J_{l}\left(\tau ,\theta \right)$ as

(29) $$J_{l}\left(\tau ,\theta \right)≜\frac{1}{f^{H}\left(\tau ,\theta \right)E_{ln}E_{ln}^{H}f\left(\tau ,\theta \right)},$$

the ranges (more specifically, the time delays associated with the ranges of targets) and angles can be jointly estimated at the *i*th radar as

(30) $$\left({\hat{\tau }}_{lk},{\hat{\theta }}_{lk}\right)=\underset{\tau ,\theta }{\arg\max}J_{l}\left(\tau ,\theta \right),\;\mathrm{for}\;k=1,\ldots ,K,$$

where *K* is the number of targets. When *K* is not known at radar, to estimate the number of signal sources, the minimum description length (MDL) [28,29] can be exploited as

(31) $${\hat{K}}_{l}=\underset{k\in \{1,\ldots ,MN\}}{\arg\min}MDL_{l}\left(k\right),$$

where

(32) $$\begin{array}{ccc} MDL_{l}\left(k\right) & = & -\log{\left(\frac{\prod_{p=k+1}^{MN}{\left(\lambda_{lp}\right)}^{1/(MN-k)}}{\frac{1}{\left(MN-k\right)}\sum_{p=k+1}^{MN}\lambda_{lp}}\right)}^{(MN-k)S}\\ & & +\frac{1}{2}k\left(2MN-k\right)\log S. \end{array}$$

Here, $\lambda_{lp}$ is the *p*th eigenvalue of ${\bar{R}}_{l}$ in (27).

## 4. Efficient High-Resolution Target Localization Algorithm with Distributed FMCW MIMO Radars

### 4.1. Target Localization Based on the Transformation of Distributed 2D MUSIC Spectra

In [20], a target localization algorithm is developed based on the coordinate transformation of distributed 2D MUSIC spectra when the distributed radars are in a straight line (i.e., $y_{l}^{r}=0$ for l=0,…,L−1). In this section, the target localization algorithm is developed based on the transformation of distributed 2D MUSIC spectra for arbitrary distributed radar positions.

**Figure 2:**
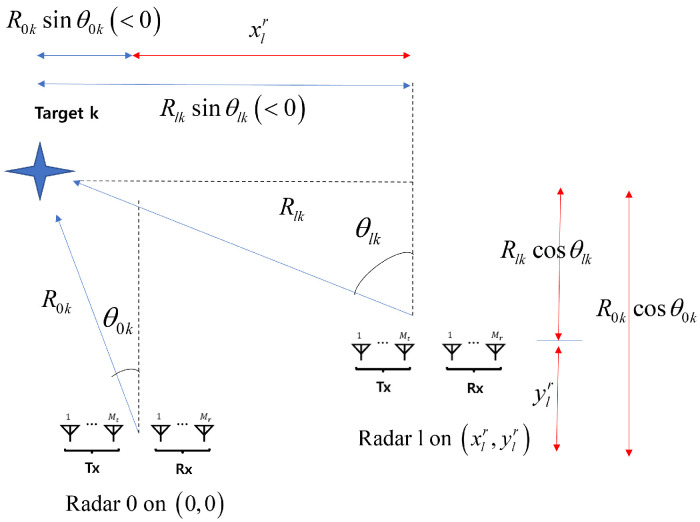
Coordinate transformation in the distributed FMCW MIMO radar system.

The range and angle pairs $\left(R_{lk},\theta_{lk}\right)$, k=1,…,K are the coordinates with respect to the *l*th FMCW MIMO radar. Accordingly, the coordinates are transformed with respect to the reference radar before the 2D MUSIC algorithm is processed. That is, rather than forwarding the raw data of the received signal to the data fusion center, each radar performs the 2D MUSIC algorithm with its own received signal using the transformed coordinates [20]. Specifically, when the *l*th radar is located in $\left(x_{l}^{r},y_{l}^{r}\right)$, from the Figure 2, we can have two equations representing the relationship between $\left(R_{lk},\theta_{lk}\right)$ and $\left(R_{0k},\theta_{0k}\right)$ as

(33) $$R_{0k}\cos\theta_{0k}=R_{lk}\cos\theta_{lk}+y_{l}^{r}$$

(34) $$R_{0k}\sin\theta_{0k}=R_{lk}\sin\theta_{lk}+x_{l}^{r}.$$

Accordingly, $\left(R_{lk},\theta_{lk}\right)$ can be expressed in terms of $\left(R_{0k},\theta_{0k}\right)$ as,

(35) $$R_{lk}=\sqrt{{\left(R_{0k}\sin\theta_{0k}-x_{l}^{r}\right)}^{2}+{\left(R_{0k}\cos\theta_{0k}-y_{l}^{r}\right)}^{2}}$$

(36) $$\theta_{lk}=\tan^{-1}\frac{R_{0k}\sin\theta_{0k}-x_{l}^{r}}{R_{0k}\cos\theta_{0k}-y_{l}^{r}},$$

Because $\tau_{lk}=2R_{lk}/c$, from (35) and (36), we can express (τ,θ) in the *l*th radar’s coordinates as,

(37) $$\begin{array}{ccc} \tau & = & \frac{2}{c}\sqrt{{\left(\frac{\bar{\tau }c}{2}\sin\bar{\theta }-x_{l}^{r}\right)}^{2}+{\left(\frac{\bar{\tau }c}{2}\cos\bar{\theta }-y_{l}^{r}\right)}^{2}}\\ & ≜ & f_{l}^{tr}\left(\bar{\tau },\bar{\theta }\right) \end{array}$$

(38) $$\begin{array}{ccc} \theta & = & \tan^{-1}\frac{\bar{R}\sin\bar{\theta }-x_{l}^{r}}{\bar{R}\cos\bar{\theta }-y_{l}^{r}},\\ & ≜ & g_{l}^{tr}\left(\bar{\tau },\bar{\theta }\right) \end{array}$$

where $\left(\bar{\tau },\bar{\theta }\right)$ is based on the reference radar’s coordinate. Then, by substituting (τ,θ) in (37) and (38) into (28) and (29), at the *l*th FMCW MIMO radar, the cost function in (29) is reformulated with respect to the reference radar’s coordinates $\left(\bar{\tau },\bar{\theta }\right)$ as,

(39) $$\begin{array}{ccc} {\bar{J}}_{l}\left(\bar{\tau },\bar{\theta }\right) & \;=\; & \;J_{l}\left(f_{l}^{tr}\left(\bar{\tau },\bar{\theta }\right),g_{l}^{tr}\left(\bar{\tau },\bar{\theta }\right)\right)\\ & \;=\; & \;\frac{1}{f^{H}\left(\left(f_{l}^{tr},g_{l}^{tr}\right)\left(\bar{\tau },\bar{\theta }\right)\right)E_{ln}E_{ln}^{H}f\left(f_{l}^{tr},g_{l}^{tr})\left(\bar{\tau },\bar{\theta }\right)\right)} \end{array}$$

Then, the distributed radars report $J_{l}\left(\bar{\tau },\bar{\theta }\right)$ for the radar image region of interest to the data fusion center. At the data fusion center, the aggregated cost function can be formulated as,

(40) $$\bar{J}\left(\bar{\tau },\bar{\theta }\right)=\sum_{l=0}^{L-1}{\bar{J}}_{l}\left(\bar{\tau },\bar{\theta }\right).$$

The delays and angles can then be jointly estimated at the data fusion center as

(41) $$\left({\hat{\bar{\tau }}}_{k},{\hat{\bar{\theta }}}_{k}\right)=\underset{\bar{\tau },\bar{\theta }}{\arg\max}\bar{J}\left(\bar{\tau },\bar{\theta }\right),\;\mathrm{for}\;k=1,\ldots ,K.$$

Then, the target location can be given as

(42) $$\begin{array}{ccc} \left({\hat{x}}_{k}^{t},{\hat{y}}_{k}^{t}\right) & = & ({\hat{\bar{R}}}_{k}\sin{\hat{\bar{\theta }}}_{k},{\hat{\bar{R}}}_{k}\cos{\hat{\bar{\theta }}}_{k}), \end{array}$$

where ${\hat{\bar{R}}}_{k}=\frac{c{\hat{\bar{\tau }}}_{k}}{2}$. Note that the distributed radars do not need to report all their measured signals to the data fusion center, but they forward their local cost function (39) for the radar image region of interest. In addition, the parameter estimation is executed at the data fusion center based on the collected MUSIC spectra.

### 4.2. Target Localization Based on the Weighted-Average of Local Estimates in a Unified Coordinate

To avoid the communication overhead for sharing the MUSIC spectra, the range/angle parameters are locally estimated at distributed FMCW MIMO radars and their estimates are forwarded to the data fusion center. That is, the target location estimated from the *l*th radar can be given as

(43) $$\begin{array}{ccc} \left({\hat{x}}_{l1}^{t},{\hat{y}}_{l1}^{t}\right) & = & ({\hat{R}}_{l1}\sin{\hat{\theta }}_{l1}+x_{l}^{r},{\hat{R}}_{l1}\cos{\hat{\theta }}_{l1}+y_{l}^{r}). \end{array}$$

From Lemma 1 and (24), the localization error due to the parameter estimation error at the *l*th radar is upper-bounded as

(44) $$\begin{array}{c} |\Delta x_{l1}^{t}|\leq \Delta x_{l,\max},\;\left|\Delta y_{l1}^{t}\right|\leq \Delta y_{l,\max}, \end{array}$$

where

(45) $$\begin{array}{ccc} \Delta x_{l,\max} & = & \frac{Res_{R}}{2}|\sin\left(\theta_{l1}\right)|+\frac{Res_{C}}{2}\left|\cos\left(\theta_{l1}\right)\right|,\\ \Delta y_{l,\max} & = & \frac{Res_{R}}{2}|\cos\left(\theta_{l1}\right)|+\frac{Res_{C}}{2}\left|\sin\left(\theta_{l1}\right)\right|, \end{array}$$

where $Res_{C}=R_{l1}Res_{\theta }$ is the cross-range resolution. Because the localization errors in (45) depend on the parameter estimates $\left(R_{l1},\theta_{l1}\right)$ at each radar, the target locations estimated from the *l*th radar are linearly combined based on the expected localization errors in (44). Specifically, by exploiting the upper bound in (45), the linearly-weighted estimate is given as

(46) $$\begin{array}{c} {\hat{x}}_{1}^{t}=\sum_{l=0}^{L-1}\alpha_{l}{\hat{x}}_{l1}^{t},\;{\hat{y}}_{1}^{t}=\sum_{l=0}^{L-1}\beta_{l}{\hat{y}}_{l1}^{t}, \end{array}$$

where

(47) $$\begin{array}{c} \alpha_{l}=\frac{1/\Delta x_{l,\max}}{\sum_{l=0}^{L-1}1/\Delta x_{l,\max}},\;\beta_{l}=\frac{1/\Delta y_{l,\max}}{\sum_{l=0}^{L-1}1/\Delta y_{l,\max}}. \end{array}$$

From (47), the estimate ${\hat{x}}_{l1}^{t}$ with smaller $\Delta x_{l,\max}$ is more contributed on ${\hat{x}}_{1}^{t}$. In addition, from (44) and (46), it can be found that the localization error of the linearly-weighted estimate can be upper bounded as

(48) $$\begin{array}{c} |\Delta x_{1}^{t}|\;\leq \;\Delta x_{\max},\;\left|\Delta y_{1}^{t}\right|\leq \Delta y_{\max}, \end{array}$$

where

(49) $$\begin{array}{c} \Delta x_{\max}\;=\;\frac{L}{\sum_{l=0}^{L\;-\;1}1/\Delta x_{l,\max}},\;\Delta y_{\max}\;=\;\frac{L}{\sum_{l=0}^{L\;-\;1}1/\Delta y_{l,\max}}, \end{array}$$

which are the harmonic means of the localization errors in distributed radars. That is, if one of the distributed radars has a small estimation error in the x-coordinate and another has a small estimation error in the y-coordinate, the overall localization error of the linearly weighted estimate can be effectively reduced.

We also note that even though individual radar processing assumes LoS, our distributed fusion approach offers significant robustness against NLoS and multipath interference due to spatial diversity effects. This is because different radars experience independent multipath environments; that is, multipath delays and angle estimates for these delays vary significantly across distributed radars. Consequently, the actual target appears at the same physical location from all radars, leading to consistent position estimates across the distributed network. In contrast, multipath reflections appear at different apparent locations from different radar positions, creating inconsistent estimates that can be filtered out. Before the weighted-averaging process at the fusion center, we can identify and filter out inconsistent estimates caused by multipath.

**Remark** **1** (Placement of distributed radars). *We note that due to the typically limited number of antennas in FMCW MIMO radars, azimuth angle resolution tends to be relatively lower than range resolution. Accordingly, range resolution is generally better than cross-range resolution. As discussed in (49), to get a more accurate localization estimate, distributed radars need to be placed such that their boresight angles to the target (or the region of interest) are orthogonal. For example, consider a scenario with two distributed radars. When M=8 and BW=250* MHz, *the range resolution is given by ${\mathrm{Res}}_{R}=0.6$* m, *and the angular resolution is ${\mathrm{Res}}_{\theta }=0.25$* rad, *as derived from (24). If the targets are located in front of the reference radar at a range of* 20 m, *then the cross-range resolution is* ${\mathrm{Res}}_{C}=R\cdot {\mathrm{Res}}_{\theta }=20\times 0.25=5.0$ m. *From (45), we obtain* $\Delta x_{0,\max}(=2.5$ m) $>\;\Delta y_{0,\max}(=0.3$ m) *when* $\theta_{01}=0$.

*Without loss of generality, we consider that $\theta_{11}\in \left[0,\frac{\pi }{2}\right]$ and the upper bounds in (49) is given as*

(50) $$\Delta x_{\max}=\frac{2\Delta x_{0,\max}\Delta x_{1,\max}}{\Delta x_{0,\max}+\Delta x_{1,\max}},$$

(51) $$\Delta y_{\max}=\frac{2\Delta y_{0,\max}\Delta y_{1,\max}}{\Delta y_{0,\max}+\Delta y_{1,\max}}.$$

*Here, $\Delta x_{\max}$ represents twice the harmonic mean of $\Delta x_{0,\max}$ and $\Delta x_{1,\max}$, and $\Delta y_{\max}$ represents twice the harmonic mean of $\Delta y_{0,\max}$ and $\Delta y_{1,\max}$. The harmonic mean is minimized when the two values are as different as possible, since the harmonic mean emphasizes smaller values [30]. In our case, this principle suggests that $\theta_{11}=\frac{\pi }{2}$ minimizes $\Delta x_{\max}+\Delta y_{\max}$.*

*In the proposed weighted averaging approach, radars with smaller estimation errors (smaller $\Delta x_{l,\max}$ or $\Delta y_{l,\max}$) receive higher weights, thus contributing more to the final estimate. The harmonic mean-based weights automatically emphasize more accurate radars, leading to improved overall localization performance. Therefore, to enhance localization accuracy, the second radar should be deployed such that $\Delta x_{1,\max}<\Delta y_{1,\max}$, which can be achieved by setting $\theta_{11}=\pi /2$.*

**Remark** **2** (Upper-bound based weighting). *The weight assignment uses deterministic upper bounds rather than statistical variance to provide worst-case performance guarantees and computational efficiency. This approach directly relates to fundamental resolution limits and provides clear geometric insight into how radar placement affects localization accuracy, making it suitable for real-time applications.*

**Remark** **3** (Synchronization and calibration uncertainties). *We note that our error analysis in Equation (20) primarily focused on parameter estimation errors. Non-idealities due to synchronization and calibration uncertainties can indeed introduce additional range and phase errors. Specifically, synchronization error contributes additional range errors such as $\Delta R_{sync}=c\Delta t_{sync}/2$, where $\Delta t_{sync}$ is a synchronization error. In addition, phase calibration uncertainties $\Delta \phi_{cal}$ can affect angle estimation accuracy. These non-idealities are incorporated into the weighting scheme by adjusting the error bounds in the weight calculation.*

*We also note that because we perform non-coherent fusion (combining position estimates rather than raw signals), the synchronization requirements are significantly relaxed. Since position estimates are incoherent, there is no need for precise phase alignment. We only require frame-level synchronization, with an accuracy of approximately 1–10* ms.

### 4.3. Communication Overhead and Computational Complexity Analysis

(Communication Overhead) We compare the communication overhead for three different data fusion methods—raw signal fusion [15,16,17], MUSIC spectrum fusion [20], and our proposed method.

For the raw signal fusion [15,16,17], all raw received signals are transmitted to the data fusion center. Specifically, each radar needs to transmit the raw I/Q data of $M_{r}\times M_{t}\times N\times S$ complex samples, where each frame consists of *S* FMCW pulses and each pulse has *N* samples. For the MUSIC spectrum fusion [20], 2D MUSIC spectra are transmitted to the fusion center. Therefore, when the MUSIC spectrum has $N_{R}\times N_{\theta }$ 2D grids points, $N_{R}\times N_{\theta }$ complex data samples need to be transmitted. In our proposed method, only target position estimates are transmitted. Each estimate (in (x,y) coordinates) takes two floating-point values, and therefore, 2K real data samples need to be transmitted for *K* targets.

For example, when eight virtual antennas with N=256 and S=256 are utilized, 8×256×256=524,288 complex samples per frame are required for the raw signal fusion. For 32 bit precision, it becomes 524,288×2×4=4.2 Mbytes per frame. When $N_{R}\times N_{\theta }=512\times 180$ with 32 bit precision, 92,160×4=369 Kbytes need to be transmitted. In contrast, when there are four targets, 32 bytes are required to be transmitted to the data fusion center. This observation is summarized in Table 1. We note that the proposed method achieves a communication overhead reduction of over five orders of magnitude compared to raw signal fusion and approximately 46,000× reduction compared to MUSIC spectrum fusion (assuming single target scenario). Accordingly, this advantage makes the proposed method suitable for the real-time applications.

(Computational Complexity) At each individual radar, the 2D MUSIC algorithm is processed, which requires $O({\left(MN\right)}^{3})$ for eigenvalue decomposition and $O(N_{R}N_{\theta })$ for spectrum computation. At the data fusion center, the proposed weighted averaging scheme takes O(L) for weight calculation/averaging with *L* distributed radars. In contrast, MUSIC spectrum fusion requires $O(L\times N_{R}N_{\theta })$ for the spectrum combination at the data fusion center. The computational complexity of the proposed method is lower than the MUSIC spectrum fusion method [20].

## 5. Simulation Results

To verify the proposed method, we perform computer simulations. In these simulations, we use a center frequency of 77 GHz and an operational bandwidth of 250 MHz. A sampling frequency of 500 MHz is applied. The number of pulses is set to 100. Additionally, the number of time samples per pulse is set to 2000. The numbers of transmit and receive antennas are, respectively, given as $M_{t}=2$ and $M_{r}=4$, where the transceiver antennas are arranged to form a virtual uniform linear array with an inter-antenna spacing of λ/2. Throughout the simulations, we assume that the line-of-sight (LoS) component is maintained for each target and there is no multi-path propagation. For the LoS path, a typical path-loss exponent of two is used; however, the model can be extended to more general types of channel model.

**Figure 3:**
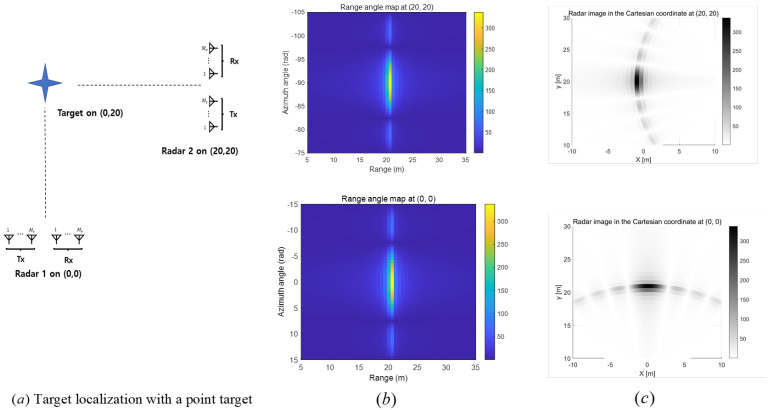
(**a**) Simulation environment with two distributed FMCW MIMO radars and (**b**) range angle maps at each radar and (**c**) Cartesian coordinate radar images when 2D DFT algorithm is utilized.

To see how the location of the distributed radars affects the target localization, we consider a scenario where the radar is positioned at (0,0) and a point target is located at (0,20), as shown in Figure 3a. First, we plot the range-angle map image in Figure 3b (lower), where the 2D DFT algorithm described in Section 3.1 is utilized. As discussed in Remark 1, the range resolution is finer than the angle resolution. In Figure 3c (lower), the corresponding radar image is presented in Cartesian coordinates. In this case, the localization error along the x-axis is expected to be larger than that along the y-axis. In contrast, Figure 3b (upper) shows the range-angle map image when the radar is positioned at (20,20). The corresponding radar image in Cartesian coordinates is provided in Figure 3c (upper), where the localization error along the y-axis is expected to be larger than that along the x-axis.

**Figure 4:**
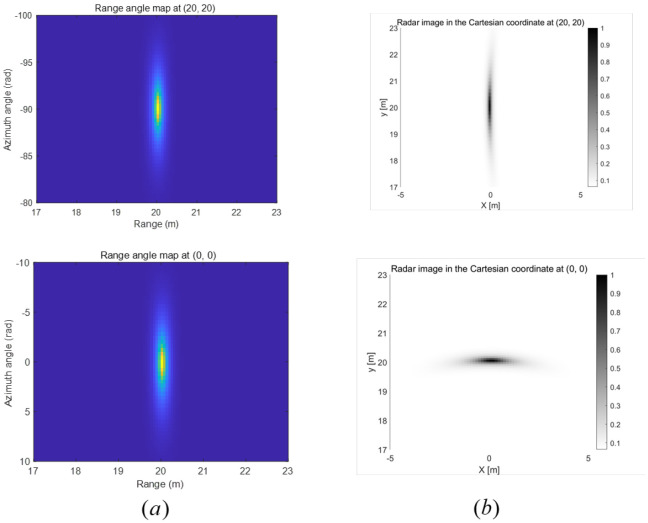
(**a**) Range angle maps at each radar and (**b**) Cartesian coordinate radar images when 2D MUSIC algorithm is utilized.

In Figure 4, we present the range-angle maps and Cartesian coordinate radar images when the 2D MUSIC algorithm, described in Section 4.1, is applied to each radar. Compared to the 2D DFT algorithm, the overall radar images obtained using 2D MUSIC exhibit higher resolution. Again, the resolution of the Cartesian coordinate radar image varies depending on the relative location of the radar with respect to the target.

**Figure 5:**
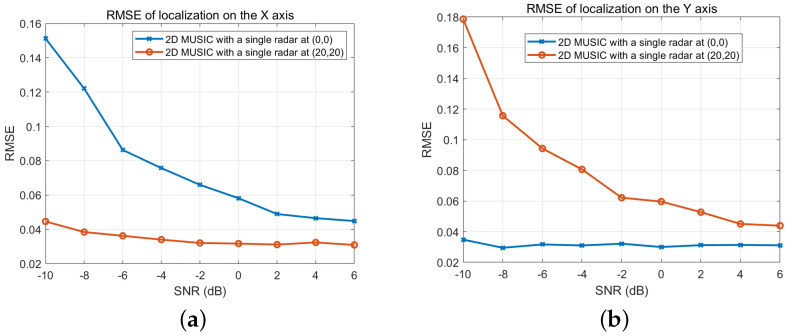
RMSE of localization along (**a**) x-axis and (**b**) y-axis.

The RMSEs of the 2D MUSIC algorithm, using the measurements from individual radars located at (0,0) and (20,20), are evaluated for various SNRs in Figure 5. In Figure 5a, the RMSEs of localization estimation along the x-axis are given. As discussed in Figure 3 and Figure 4, the radar located at (0,0) exhibits better RMSE performance than the radar at (20,20) in terms of x-axis localization accuracy. In Figure 5b, the RMSEs of localization estimation along the y-axis are shown. In this case, the radar at (20,20) demonstrates better RMSE performance than the radar at (0,0) for y-axis localization accuracy.

**Figure 6:**
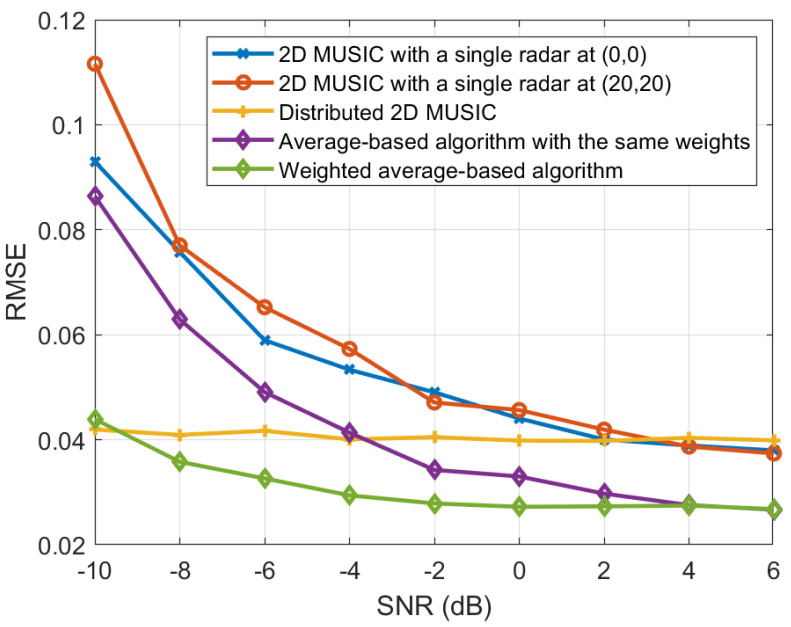
RMSE comparison for various localization algorithms.

The RMSEs of the proposed weighted average-based localization estimation are evaluated for various SNRs in Figure 6. For comparison, we also present the RMSEs of the 2D MUSIC algorithm using the measurements from individual radars located at (0,0) and (20,20), respectively. In addition, we evaluate the RMSE of target localization based on the transformation of distributed 2D MUSIC spectra, as described in Section 4.1, which requires transmitting the MUSIC spectra from each radar to the data fusion center. Furthermore, the RMSEs of the average-based estimate with equal weighting are also analyzed. As shown in the figure, the proposed weighted average-based localization algorithm achieves the lowest RMSE. This is because it effectively exploits the spatial diversity to reduce localization errors by assigning different weights based on the radar locations. Interestingly, when the SNR is low, localization performance is more affected by noise. Consequently, the performance gap between the weighted averaging method and the equal-weight averaging method becomes more pronounced.

**Figure 7:**
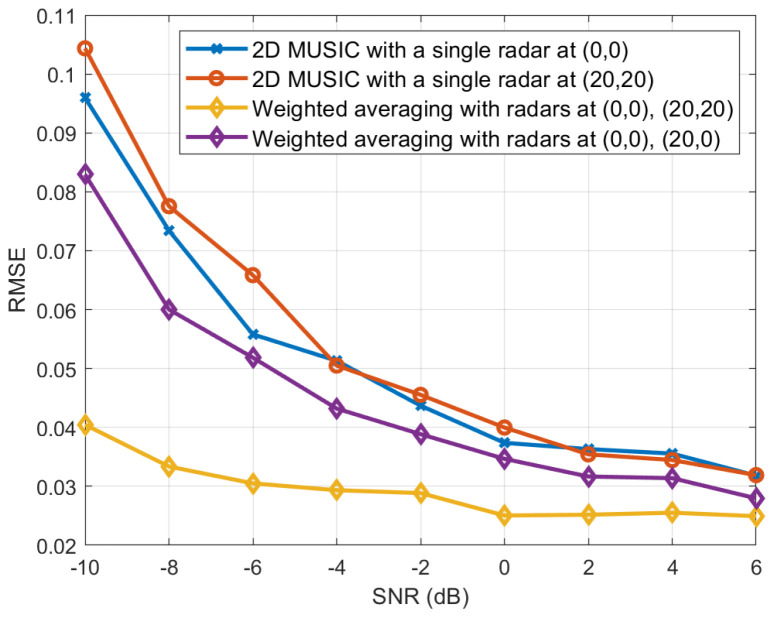
RMSE of the proposed weighted average-based algorithm for two different placements of two distributed radars.

In Figure 7, RMSEs are evaluated for two different placements of two distributed radars. Specifically, in the first case, two radars are positioned at (0,0) and (20,20), while in the second case, they are placed at (0,0) and (20,0). The proposed weighted average-based localization algorithm is then applied. From the figure, it can be found that the radar placement at (0, 0) and (20, 20) results in better localization performance compared to the placement at (0, 0) and (20, 0). This is due to the fact that the reference radar at (0, 0) has a relatively small estimation error along the x-axis. Consequently, when the second radar exhibits a small estimation error along the y-axis (i.e., when $|\theta_{01}-\theta_{11}|\approx \frac{\pi }{2}$), the overall estimation error of the weighted average-based localization algorithm is minimized. This result is consistent with the discussion in Remark 1.

To see the effect of the weighted-averaging and the placement of distributed radars on the estimation performance, we only focus on a single target in the experiment, but the proposed method can be consistently extended to multi-target environments. As the reviewer pointed out, we agree that the data association becomes a critical issue in multi-target environments. One simple but effective method is the distance-based association with gating, where estimates are associated if their coordinate distances are below a predefined threshold.

## 6. Experiment Results

**Figure 8:**
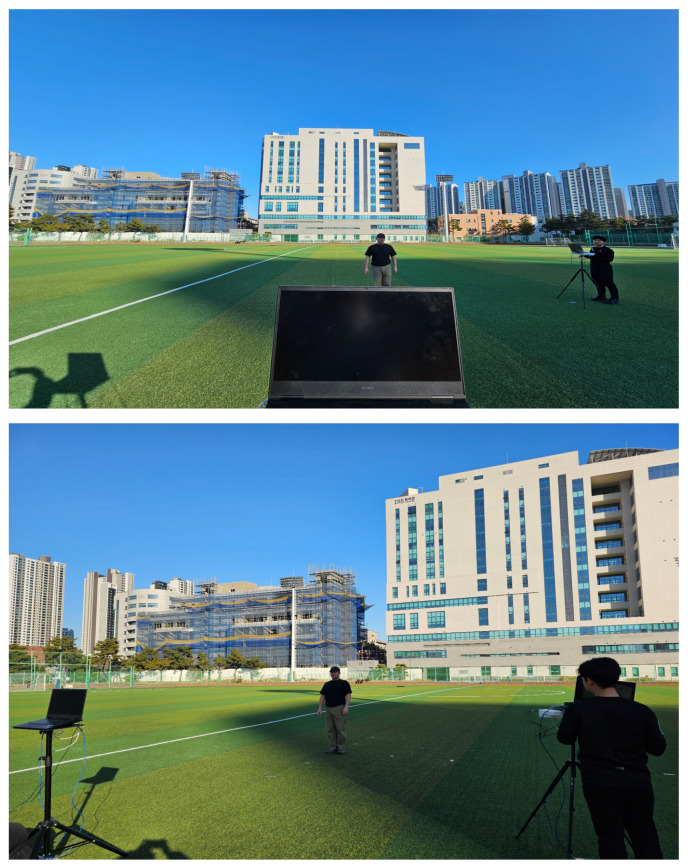
Field experiment setup.

The proposed algorithm is demonstrated through an experiment using two W-band FMCW MIMO radars (TI AWR-1642-BOOST) equipped with 2 Tx and 4 Rx antennas, as shown in Figure 8. The FMCW chirp configuration used in our experiment is provided in Table 2.

**Figure 9:**
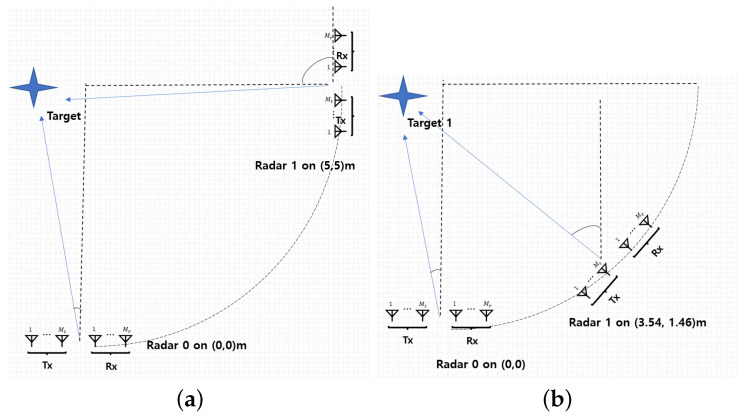
Experiments with two different radar deployment scenarios: (**a**) two radars located at (0, 0)m and (5, 5)m (**b**) two radars located at (0, 0)m and $\left(5\;\sin\pi /4,\;5\left(1-\cos\pi /4\right)\right)m\left(\;=\;3.54,\;1.46\right)$.

The experiments are conducted with two different radar deployment scenarios, as shown in Figure 8. In the first scenario, the two radars are positioned at (0, 0)m and (5, 5)m, as shown in Figure 9a. In the second scenario, they are located at (0, 0)m and $\left(5\sin\pi /4,\;5(1-\cos\pi /4)\right)$m, which is approximately $\left(3.54,\;1.46\right)$ in Figure 9b. For each deployment scenario, experiments are carried out with varying target positions near (0, 5)m.

**Figure 10:**
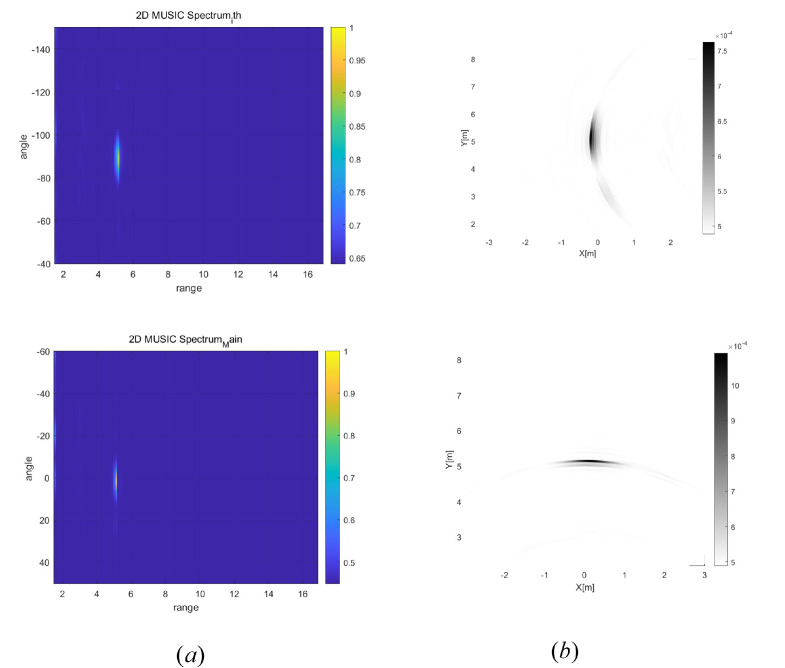
(**a**) Range angle maps at each radar and (**b**) Cartesian coordinate radar images when 2D MUSIC algorithm is applied to the experimental data collected at the distributed radars.

In Figure 10, the range-angle maps and the corresponding radar images in Cartesian coordinates are evaluated when the 2D MUSIC algorithm is applied to the de-ramped baseband signals collected from distributed radars positioned at (0, 0)m and (5, 5)m, respectively. Here, a single target is located at (0, 5)m. In Figure 10a (lower), the range-angle map for the radar at (0, 0)m is shown. Here, the range resolution is finer than the angle resolution. Accordingly, in the associated radar image in Figure 10b (lower), the resolution along the x-axis be higher than that along the y-axis. In contrast, in Figure 10a (upper), the range-angle map for the radar at (5, 5)m is presented. Additionally, in Figure 10b (upper), the corresponding radar image in Cartesian coordinates shows that the localization error along the y-axis is expected to be greater than that along the x-axis. Therefore, as observed in the simulation results shown in Figure 3 and Figure 4, the experimental results confirm that the resolution of the Cartesian coordinate radar image varies depending on the relative location of the radar with respect to the target.

To confirm this observation, in Table 3, we evaluate the RMSE when a target is placed at four different locations: (0, 5)m, (0, 4)m, (0, 6)m, and (1, 5)m. For each target location, 50 trials are conducted to obtain the RMSE in Cartesian coordinates. From the table, it is observed that the radar at (0, 0)m has a lower RMSE along the y-axis compared to the radar at (5, 5)m. In contrast, it has a higher RMSE along the x-axis than the radar at (5, 5)m.

In Table 4, the RMSEs of the weighted average based localization algorithm under two different deployment scenarios are evaluated. For comparison, the RMSEs of the 2D MUSIC algorithm using a single radar, as well as the equal-weight combining method with two distributed radars, are also assessed based on the experimentally measured data. The results demonstrate that RMSE performance improves when multiple radars are utilized. This is because the low cross-range resolution, caused by the limited number of antennas in each radar module, degrades the RMSE performance. However, this issue can be mitigated by leveraging distributed radars. Furthermore, the proposed weighted averaging approach outperforms the equal-weight combining method. By assigning weights proportional to the achievable parameter resolution, the estimation error is further reduced. Additionally, it is confirmed that the placement of the distributed radars significantly impacts RMSE performance. Specifically, the deployment at (0, 0) and (5, 5) provides better performance compared to the placement at (0, 0) and (3.54, 1.45).This can be explained by the fact that the reference radar at (0, 0) has a relatively small estimation error along the x-axis. Accordingly, when the second radar exhibits a small estimation error along the y-axis (i.e., when $|\theta_{01}-\theta_{11}|\approx \frac{\pi }{2}$), the overall estimation error of the weighted average-based localization algorithm is minimized, which is consistent with the discussion in Remark 1.

Note that the simulation parameters (250 MHz bandwidth) differ from the experimental setup (1279.2 MHz bandwidth) to demonstrate algorithm generalizability across different system configurations. The experimental validation confirms real-world applicability using actual radar hardware parameters, while the simulations explore broader parameter ranges for comprehensive performance analysis. Furthermore, in our experiment, we focused on a single target to isolate the effects of weighted-averaging and the placement of distributed radars on estimation performance. However, our proposed method can be consistently extended to multi-target environments. In this setting, data association becomes a critical issue. A simple but effective approach is distance-based association with gating, where estimates are associated if their coordinate distances are below a predefined threshold.

## 7. Conclusions

In this paper, we have proposed an efficient high-resolution target localization algorithm for distributed FMCW MIMO radar systems. Unlike conventional methods that require transmitting raw received signals or MUSIC spectra to a data fusion center, our approach significantly reduces communication overhead by forwarding only target position estimates from individual radars. These estimates are then weighted and averaged in a unified coordinate system at the data fusion center, where the weights are assigned based on the achievable resolution of range and angle estimation. Through a detailed analysis, we demonstrated that localization errors in the unified coordinate system depend on the placement of distributed radars and the local estimation accuracy of range and angle parameters. Our method effectively addresses the limitations of FMCW MIMO radars, which typically suffer from lower azimuth angle resolution due to a limited number of antennas. By leveraging multiple distributed radars and appropriately weighting their estimates, we significantly enhance localization accuracy while maintaining computational efficiency. The effectiveness of the proposed algorithm was validated through both synthetic simulations and real-world experimental measurements using two W-band FMCW MIMO radars. The results confirmed that our approach achieves superior localization performance compared to conventional methods while reducing implementation complexity. Additionally, we analyzed how different radar placements affect localization performance, highlighting the importance of optimizing radar deployment in distributed radar networks.

## Figures and Tables

**Table 1:** Communication Overhead comparison when 8 virtual antennas with N=256 and S=256 are utilized with 32 bit precision.

Method	Data per Frame	Reduction Factor
Raw Signal Fusion [15,16,17]	4.2 MB	1
MUSIC Spectrum Fusion [20]	369 KB	11.4×
Proposed Method	8 bytes/target	525,000×

**Table 2:** FMCW experiment parameter setting.

Parameter	Value
Type of Signal waveform	Linear Chirped waveform
Chirp BW	1279.2 (Mhz)
Number of Chirps per frame	256
Number of Chirp Loops	128
Range resolution	0.1172 (m)
Velocity resolution	0.0470 (m/s)
Tx power	12 dBm
Sampling Rate	6000 ksps

**Table 3:** RMSE of localization on x-axis and y-axis with experimental data.

RMSE	Target Locations			
(*ϵ_x_*,*ϵ_y_*)	(0, 5)m	(0, 4)m	(0, 6)m	(1, 5)m
Radar at (0,0)m	(0.375,0.073)	(0.239,0.063)	(0.201,0.144)	(0.163,0.107)
Radar at (5,5)m	(0.064,0.210)	(0.103,0.281)	(0.105,0.259)	(0.049,0.263)

**Table 4:** RMSE of localization algorithms with two different deployment scenarios.

RMSE	Target Locations			
	(0,5) m	(0,4) m	(0,6) m	(1,5) m
2D MUSIC with a single	0.224	0.151	0.173	0.135
radar at (0,0)m				
2D MUSIC with a single	0.137	0.111	0.192	0.149
radar at (5,5)m				
Equal-weight combining with	0.130	0.083	0.192	0.130
radars at (0,0)/(3.54,1.45)m				
Equal-weight combining with	0.149	0.110	0.011	0.076
radars at (0,0)/(5,5)m				
Proposed weighted averaging with	0.115	0.060	0.127	0.085
radars at (0,0)/(3.54,1.45)m				
Proposed weighted averaging with	0.069	0.057	0.094	0.044
radars at (0,0)/(5,5)m				

## Data Availability

Data are contained within the article.
